# Aims to Reduce Coercive Measures in Forensic Inpatient Treatment: A 9-Year Observational Study

**DOI:** 10.3389/fpsyt.2020.00465

**Published:** 2020-05-27

**Authors:** Steffen Lau, Nathalie Brackmann, Andreas Mokros, Elmar Habermeyer

**Affiliations:** ^1^Department of Forensic Psychiatry, University Hospital of Psychiatry Zurich, Zurich, Switzerland; ^2^Department of Psychology, Fern Universität in Hagen, Hagen, Germany

**Keywords:** prevention of torture, forensic psychiatry, seclusion, restraint, schizophrenia

## Abstract

Protecting the human rights is particularly important within the forensic context because patients in forensic psychiatry are not admitted voluntarily and so the treatment itself is of a coercive nature. Coercive measures (*i.e.*, actions against the will of the patient such as forced medication, seclusion or restraint) form an additional incision of personal rights. Although the use of coercion within forensic psychiatric institutions remains controversial, little empirical research has been conducted on the use of coercive measures within forensic settings. The study presented here can contribute to close this research gap by informing about rates of coercive measures within the present institution. National and international organizations on the prevention of torture or inhuman or degrading treatment have emphasized the need to keep the incidents of coercive measures to a minimum. Criticisms by such organizations on high rates of seclusion, restraint, and compulsory medication have led to organizational changes within the present institution which is Switzerland’s largest forensic clinic with an average of 124 patients per year. After a first visit of such a committee, *e.g.*, the detailed documentation of coercive measures became obligatory and part of special reports. Changes in the use of coercive measures are presented here. Data on coercive measures was analyzed for years 2010 to 2018. With respect to the most invasive coercive measurement, restraint, a minimum of four patients in 2017 and a maximum of 14 patients in 2010 have been subject to this form of coercive measurement. A minimum of sixteen patients in 2012 and a maximum of 40 patients in 2010 were secluded. Though total number and duration show a trend towards a reduction in severity of coercive measures on average, a few patients are not responsive to deescalating interventions. Preventive mechanisms, documentation standards, and efforts to ensure humane and adequate treatment are discussed under ethical considerations of coercive measures within court mandated treatment.

## Introduction

It is an essential principle in medical ethics that patients should be left to make their own choices (1). The freedom of choice and consent is challenged in secure psychiatric care. Patients detained under mental health legislation are impaired in decision making. Forensic psychiatric detainment is in itself of coercive nature. Treatment in forensic settings is justified by reasons of public safety; a patient’s right for autonomy is considered less important than public security and safety. Compulsory treatment (*i.e.*, actions against the will of the patient such as forced medication, seclusion, or restraint (2) forms an additional incision of personal rights. Forensic psychiatrists may compel patients into taking medication that is intended to reduce their risk of behaving violently but may also be used as a chemical restraint (3) to reduce a patient’s capacity of moving around. These measures are considered as necessary in the management of dangerous behavior against self or others, though the use of coercive measures can be accompanied by adverse side effects including traumatization of patients and staff (4, 5).

To address the complex problem, some official European Organizations or multicentric approaches (*e.g.*, the EUNOMIA study) have tried to develop and evaluate guidelines (6, 7). For an expert consensus on how to deal with agitation, see (8). It was emphasized that the use of coercive measures for prolonged periods should be reserved to only exceptional cases. Especially in general psychiatry, low rates of coercive interventions have been described as an indicator of a high quality of psychiatric treatment (9).

In the last decades efforts were made to better understand the phenomenon by identifying influencing factors on rates of coercive measures and to reduce coercion within psychiatry (10). In general psychiatry in Europe and North America, the percentage of patients exposed to coercive measures ranges from 0 to 23% (10, 11). A diagnosis of psychotic disorder and personality disorder, substance-use related disorders, and mental retardation was identified to increase risk for experiencing coercive measures [for an overview see (12)]. Furthermore a history of aggression and threats as well as agitation and disorientation was found to be associated with the use of coercion as well as a history of former involuntary admissions and repetitive or longer hospitalizations (13, 14). Gender and age are controversially associated with rates of coercive measures: some studies have identified women to be at higher risk of coercion (14), others men, and/or younger patients (13, 15), while others have found no relationship between gender nor age (16).

Inconsistencies between studies may be caused by differences in treatment culture, organizational factors, different legislation in different countries, and societal factors as well as different methodological approaches and/or different definitions used (11, 17). Forced medication, for instance, is restricted in the Netherlands, and mechanical restraint is highly uncommon in the UK (10, 18). Additionally, for example the term “coercion” has different or overlapping definitions in the literature. The common denominator in the definition of restraint, for instance, is the reduction of one person’s ability to freely walk around. This may be realized by determining one person’s whereabouts, staff holding the person that is being restrained, or putting a device on the person that ensures the restriction of movements (*e.g.*, belts on a bed) (3). There is also no clear consensus on “how” to restrain—meaning that different areas of the body may be fixated. Sometimes enforced medication is also considered to be a form of restraint as it chemically impacts a person’s responsiveness. Furthermore, studies differ in the design used to evaluate coercion within treatment processes (by questionnaires handed out to staff or patients, extracting data from official reports *etc*.). To our knowledge there is no longitudinal analysis of the frequency of coercive measures within forensic psychiatric services in Switzerland. Therefore it is one aim of the present study to provide such data.

Empirical research on the use of coercive measures within forensic psychiatry is growing but still not as extensive as in general psychiatry. A systematic review conducted in 2013 reported varying rates, frequencies, and durations of restraint and seclusion in a range from 27.7 to 40.0% in forensic wards (19). In this review females were more likely to be restrained or secluded than males, but males tended to be restrained for longer periods than females. Younger patients tended to be secluded more often than older patients.

The European Committee for the Prevention of Torture and Inhuman or Degrading Treatment or Punishment (CPT) visits facilities in varying European countries each year and publishes detailed reports. Till now, 456 visits in total with approximately 18 visits per year were carried out in the 47 member states of the Council of Europe. During those visits, the CPT delegation receives unrestricted access to the respective institution. The CPT also published a total of 413 visit reports with findings, recommendations, comments, and requests for information (as of March 2020, www.coe.int/en/web/cpt/home). In 2011 a first visit by members of the CPT was performed within the present inpatient forensic-psychiatric institution. This visit initiated changes in the institution which were accelerated by another visit by the Swiss National Commission for the Prevention of Torture (NCPT, in German: Nationale Kommission zur Verhütung von Folter, NKVF) in 2012. Both visits resulted in criticism about high rates of coercive measures so the responsible Health Administration of the Canton of Zurich placed the order to make efforts to reduce those interventions. The integration of the present institution into the organization of a larger university clinic gave the opportunity to use already established procedures and processes to be applied within the present forensic facility. Those changes included:

- obligation to follow guidelines
- establishment of detailed documentation about interventions, to control the process of the coercion order (responsible physician, controlling nursing staff, frequency of control visits, reports of patient’s condition, detailed risk assessment, and documentation),
- accompanying the patient in restraint continuously
- an increase in the frequency of control visits to the patient to assess whether the coercive measures are still legitimated from twice daily to every two hours,
- enforcing the staff to use other strategies to avoid coercion, such as intensifying one-on-one care, and
- mandatory special trainings in de-escalation of all nursing and medical staff at the beginning of their employment and yearly fresh-ups.

This change process was accompanied by regular reports to the Health Administration and the Legal Administration of the canton. Those reports build the source for the data presented here.

This article seeks to inform the reader about special circumstances within a forensic psychiatric facility. There is limited empirical research on the use of coercive measures in forensic psychiatric institutions. Our primary aim is to address this research gap by informing about rates of coercive measures within the present institution. As to our knowledge, we are the first to report longitudinal data on the use of coercive measures within a forensic psychiatric institution in Switzerland and for a period of nine years. Lastly, we will evaluate trends in the use of coercive measures to assess whether the implemented changes have led to the intended reduction.

## Method

The current study describes 9-year follow-up data (2010–2018) on coercive measures (seclusion, restraint, and forced medication) of a single mental health facility; a forensic psychiatric institution specialized in the treatment of patients suffering from schizophrenia or other acute psychiatric pathology. The clinic offers a total of 79 beds (92 since October 2018). With an average of 124 patients per year, it is Switzerland’s largest forensic clinic. The clinic offers court mandated treatment for patients who have committed a crime or regular prisoners whose mental health status does not allow treatment within prison. 27 of the beds are according to “The Matrix of Security” (20, 21) within a high security setting, 39 (52 since October 2018) are on closed wards with medium to low security level, and 13 on an open ward with low security level.

The use of coercive measure is legislated within both health and penitentiary legislation (Swiss Civil Code, Zurich Patient Act, and Zurich Penitentiary Ordinance). Coercive measures are permitted if the patient poses a high risk of injury to him-/herself or to others, and this risk cannot be managed by less invasive measures. This order must be posed by a physician, be controlled bihourly, and the patient must be informed about his right to appeal against the order. The use of coercive measures must be reported to the head physician and the head nurse. In the present institution, seclusion is considered as the placement of a patient alone in a locked room that has been specifically designed for this purpose. Restraint is practiced as mechanical restraint, where a device is used to fixate a patient (*e.g.*, a belt). Both measures are used to restrict a patient’s capacity to move. Involuntary or forced medication is meant as the administration of a pharmacologically effective substance against a patient’s will by intramuscular injection.

For our analysis we only considered coercive measures that were put into place because of individually assessed risk of harm. Instances in which patients had been locked into their room because of organizational reasons (*e.g.*, major constructional work, overnight) are not taken into account.

### Design and Procedure

This study is a longitudinal, observational dynamic cohort study. The clinic is legally obliged to document each instant of coercive measure. Ethical approval was sought and it was decided by the local ethics committee that the study does not fall within the Human Research Act (BASEC-Nr. Req-2019-00550). Therefore, there is no need for ethical approval.

Responsible for the documentation on paper and justification are, initially, the assistant physician and a member of the nursing staff. Subsequently, the coercive measure is validated by the nurse responsible for the ward, the head nurse, the senior physician, and the head physician. The paper documents are digitalized by administrative staff.

### Sample

The total patient population per year and information on the duration of the stay are depicted in **Table 1**. Note that these are all patients that have been treated in the clinic within the respective year and not the number of patients that has been subject to at least one coercive measure (see *Results* section for the latter). More than 90% of the patients were treated for schizophrenia. Comorbidity rates were high with an average of two-thirds of patients per year having more than one diagnosis, substance related disorders being the most frequent secondary diagnosis (roughly 90% of the secondary diagnoses). Patients were treated for an average of two years (Median = 1.3 years). The cohorts are comparable with respect to the number of treated patients (ranging from 118 to 128), gender ratio (the female:male ratio ranging from 1.03:10 to 1.81:10), and diagnoses (more than 90% of patients suffering from schizophrenia).

**Table 1:** Total number of patients treated within the forensic institution.

	2010	2011	2012	2013	2014	2015	2016	2017	2018
Total number of patients	125	128	118	119	131	124	123	123	123
Female (%)	–	12 (9.4)	14 (11.9)	17 (14.3)	16 (12.2)	19 (15.3)	12 (9.8)	13 (10.6)	16 (13.0)
Male (%)	–	116 (90.6)	104 (88.1)	102 (85.7)	115 (87.8)	105 (84.7)	111 (90.2)	110 (89.4)	107 (87.0)
Mean duration of stay (in days)	896	898	842	779	671	670	667	724	670
Median duration of stay (in days)	613	529	530	433	336	396	512	483	502

### Data Preparation and Analyses

The measures of interest are the type of coercive measurement (seclusion, restraint, or forced medication) and, for seclusion and restraint, the duration of the specified action (including starting and ending point). These measures are aggregated on a yearly level for a timeframe of twelve months (January 1 to December 31 of the respective year). More specifically, we calculated the total number of coercive measures, number of patients, maximum number of incidents per patient, and, for restraint and seclusion, percentage male/female, minimum/maximum duration per incident and accumulated over the year. If not mentioned otherwise, data refers to patients that were subject to at least one incident of coercive measurement (not across total patient population). Once a patient is restrained, he or she is also considered to be secluded. This is because restraint in the current facility is applied in specially designed rooms on every ward which are locked after the initiation of restraint with a staff member accompanying the patient continuously. Measurements on seclusion and restraint are therefore not independent from each other. Forced medication may also be accompanied by another form of coercive measure (*i.e.*, seclusion and/or restraint).

To test for trends over the time period of nine years, we performed linear regressions using the least square method with year as independent variable. We chose linear regression because of our prediction (positive effect of our policy change), though different models might have a better model fit.

## Results

**Table 2** shows an overview of seclusions in years 2010 to 2018. The data reveals fluctuations in the total number of seclusion (measured per incident) over the years. No time sensitive changes are apparent here, though there is a trend toward higher numbers from year 2014 onwards, with a peak in 2018 (273 single incidents). This counterintuitive result is qualified by the number of patients in seclusion which has reduced by almost half (46.7%) between years 2011 and 2012 and stayed roughly constant between years 2012 and 2018 (with an average of 20 patients in seclusion per year).

**Table 2:** Seclusions in years 2010 to 2018.

	2010	2011	2012	2013	2014	2015	2016	2017	2018
Total number of seclusion	54	74	35	63	111	139	137	96	273
Mean duration in seclusion	126:57	75:40	35:24	5:36	4:37	13:14	5:30	6:01	13:18
Median duration in seclusion	75:37	20:30	7:00	1:30	2:00	2:00	2:05	4:07	13:00
Number of patients in seclusion	40	30	16	18	19	24	20	20	23
Female (%)	4 (10.0)	6 (20.0)	3 (18.8)	4 (22.2)	3 (15.8)	6 (25.0)	2 (10.0)	1 (5.0)	4 (17.4)
Male (%)	36 (90.0)	24 (80.0)	13 (81.2)	14 (77.8)	16 (84.2)	18 (75.0)	18 (90.0)	19 (95.0)	19 (82.6)
Max. number per pat. in seclusion	5	15	10	13	30	52	36	29	132
Min.-duration in seclusion	1:15	0:25	0:45	0:15	0:25	0:15	0:05	0:20	0:10
Max.-duration in seclusion	439:45	744:30	322:45	49:30	43:25	1177:30	47:00	14:10	684:39
Min.-duration per pat./year in seclusion	1:15	1:00	1:40	1:12	2:00	0:45	1:00	0:20	1:30
Max.-duration per pat./year in seclusion	828:30	1032:45	352:55	73:45	118:32	1241:45	482:50	327:09	1612:48
Mean duration per pat./year in seclusion	171:23	169:48	78:41	19:37	25:08	79:27	37:40	28:54	158:00
Median duration per pat./year in seclusion	106:45	82:03	41:00	14:20	13:40	11:00	8:37	4:45	14:53

Durations are reported in the format hhh:mm.

After the policy change in 2011, there is also a somewhat constant decrease in the duration of seclusions per incident as well as in total per patient and year. Surprisingly, the year 2018 is again an outlier with comparably high median and mean durations. A closer examination of the raw data revealed two outliers, meaning that these patients had repeated and long-lasting incidents of seclusion. One patient was secluded 132 times and the total duration accumulated to 1,612 h (2 months and 6 days). Another patient was secluded 68 times (total duration 656 h/27 days). Only two patients accounted for 200 and therefore 73% of all incidents of seclusion in 2018.

To account for these outliers, we performed linear regressions only for median durations. The slopes showed an overall negative trend of seclusion in median duration per incident (F(1,7) = 3.73, *p* = .095, R^2^ = .35, using y = −5.15x + 39.95) and per patient and year (F(1,7) = 15.24, *p* = .006, R^2^ = .69, using y = -11.12x + 88.63), though only the last model reached significance.

As can be seen from **Table 3**, there is also no consistent pattern with respect to the total number of restraints over the years. There is a minimum of 6 restraints in 2017 and a maximum of 69 in 2018. We again see outliers for year 2018. The same patients as mentioned in the section about seclusion also were restrained more often and longer than other patients. One of these patients was restrained 23 times with an accumulated duration of 95 h (~4 days), and the other patient was restrained 34 times, which added up to roughly 342 h over the year (14 days). The maximum duration of a single event in restraint in 2018 was 28½ days (~684 h). This is comparable to the total maximum over the years in 2011, where a patient had been secluded for 706 h. There is another peak in 2015 where a patient had been restrained 43 times, accounting for 64% of all restraints in this year. Notwithstanding these outliers in 2015 and 2018, we see a slight trend towards shorter durations after policy change in 2011 with a minimum in almost all endpoints in 2017.

**Table 3:** Restraint in years 2010 to 2018.

	2010	2011	2012	2013	2014	2015	2016	2017	2018
Total number of restraints	22	35	13	23	31	67	14	6	69
Mean duration in restraint	98:44	56:06	25:05	2:42	5:02	10:56	10:22	4:01	18:35
Median duration in restraint	90:00	8:13	10:40	1:00	2:13	1:30	2:45	1:25	4:30
Number of patients in restraint	14	13	8	8	12	10	7	4	9
Female (%)	1 (7.1)	1 (7.7)	2 (25.0)	3 (37.5)	2 (16.7)	2 (20.0)	1 (14.3)	1 (25.0)	3 (33.3)
Male (%)	13 (92.9)	12 (92.3)	6 (75.0)	5 (62.5)	10 (83.3)	8 (80.0)	6 (85.7)	3 (75.0)	6 (66.7)
Max. number per pat. in restraint	4	15	3	9	8	43	6	3	34
Min.-duration in restraint	1:15	0:25	1:00	0:05	0:45	0:31	0:30	1:00	0:13
Max.-duration in restraint	284:00	706:15	137:00	18:55	43:25	595:00	47:00	13:30	684:39
Min.-duration pat./year in restraint	1:15	3:20	1:00	0:15	1:00	1:40	2:20	1:00	0:13
Max.-duration pat./year in restraint	284:00	706:15	137:00	18:55	43:25	600:10	116:30	20:14	684:39
Mean duration pat./year in restraint	155:08	151:20	40:46	7:45	13:01	73:18	20:45	6:01	142:31
Median duration pat./year in restraint	141:52	90:20	24:25	6:43	7:15	5:15	3:40	1:25	18:35

Durations are reported in the format hhh:mm.

The analysis of regression showed, as in seclusion, an overall negative trend of restraint in median per incident (F(1,7) = 3.89, *p* = .089, R^2^ = .36, using y = −6.30x + 45.06) and per patient and year (F(1,7) = 8.68, *p* = .021, R^2^ = .55, using y = −13.38x + 100.18), though again only the last model reached significance.

A reduction in forced medication is not apparent from the data (as presented in **Table 4**). Data ranges from a minimum of nine episodes of forced medications in 2012 to a maximum of 16 episodes in 2014. In 2013, one patient was receiving medication against his/her will 97 times. It is not possible from our data to delineate what kind of medication has been administered to the patients.

**Table 4:** Forced medication in years 2010 to 2018.

	2010	2011	2012	2013	2014	2015	2016	2017	2018
Number of patients subjected to forced medication	10	12	9	12	16	15	13	12	11
Female (%)	1 (10.0)	3 (25.0)	1 (11.1)	2 (16.7)	3 (18.8)	2 (13.3)	0 (0.0)	0 (0.0)	3 (27.3)
Male (%)	9 (90.0)	9 (75.0)	8 (88.9)	10 (83.3)	13 (81.3)	13 (86.7)	13 (100.0)	12 (100.0)	8 (72.7)
Maximum number per patient	2	45	4	97	9	15	9	5	20
Median number	1	1	1	1	2	2	1	2	3

**Figure 1:**
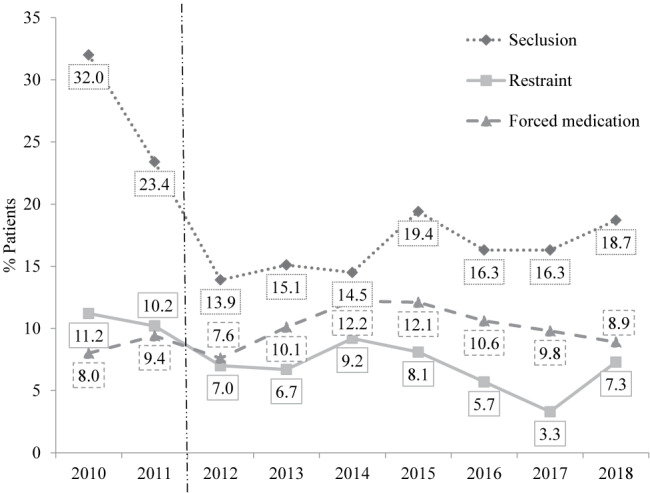
Percentage of patients subjected to at least one incident of coercive measure (relative to the total patient population). The dotted vertical line represents the policy change.

The graphs in **Figure 1** illustrate the percentage of patients that have been subjected to at least one incident of coercive measure. Note that these data are relative to the total patient population while the data above only refers to those patients that have been subjected to at least one incident of the respective coercive measure. From the data in the figure it is apparent that before the policy change in 2011, seclusion was used most often, followed by restraint, and lastly by forced medication. This order changed after the new policy was established. From 2012 onwards it is, relatively speaking, still most likely to use seclusion as coercive measure, but this is followed by forced medication and not restraint. Therefore the relative likelihood of using forced medication increased while restraint got least likely as a coercive measure.

Overall, a negative trend is apparent in percentage of patients in seclusion (F(1,7) = 2.58, *p* = .152, R^2^ = .27, using y = −1.09x + 24.29) and restraint (F(1,7) = 7.31, *p* = .030, R^2^ = .51, using y = −0.63x + 10.76), while the trend only reached significance in restraint. No negative trend was found in forced medication (F(1,7) = 1.06, *p* = .338, R^2^ = .13, using y = 0.21x + 8.79).

## Discussion

A lack of empirical research on the use of coercive measures within forensic psychiatry was stated. To our knowledge this is the first detailed description of use and changes of coercive measures over a period of several years within an inpatient forensic-psychiatric institution. To what extent the results of the study can be applied to patient populations in other states/countries than Switzerland, *e.g.*, due to legal differences, would have to be evaluated by replicating the survey in other institutions and countries. The aim was to analyze the use of coercive measures with respect to prevalence, frequency, and duration from data obtained for official reports.

Seclusion was most often applied followed by restraint and forced medication (but see years 2010 and 2011). This relative frequency of the use of coercive measures is in accordance to earlier findings (22). Compared to empirical findings, the amount of patients who experience coercive measures in our study is in the lower range of frequencies reported (23). In one study from Germany, for example, up to 31.4% of patients were affected by seclusion and up to 9.3% by restraint (24). It has to be considered that the present institution is specialized in the treatment of patients with schizophrenia. Among the general psychiatry population patients diagnosed with schizophrenia are known to exhibit higher rates of seclusion and restraint (25). In a representative German sample for inpatient psychiatric care (N = 36,690 cases), 9.5% of all patients were subjected to some kind of coercive measure. This number was significantly higher for schizophrenic patients (16.1%). One publication comparing general and forensic psychiatry in Southern Germany showed for forensic patients suffering from schizophrenia a percentage of up to 29% experiencing seclusion and around 5% experiencing restraint (26).

Our data shows a decrease from 2011 to 2017 in two of the three domains which were investigated (in seclusion and restraint, but not in forced medication) with an increase again in 2018. This last increase might be explained by the relatively seldom occurrence of coercive measures and the small number of patients who were affected. Minor fluctuations in the frequency of coercive measures might therefore be pronounced. Though total number and duration show a trend towards a reduction in severity of coercive measures on average, a few patients are not responsive to deescalating interventions. Several studies have indicated that only a few patients cause the majority of violent incidents in hospitals (27–30).

As mentioned above, we observed a change in the order that coercive measures were used. While it was more common to resort to restraint than to forced medication in the years 2010 and 2011, this relative favoritism changed to restraint being the least likely coercive measure in the years thereafter. This might reflect differences between the staff’s ethical considerations towards coercive medication and restraint. The new policy had labeled restraint as the most invasive intervention in accordance with psychiatric tradition in Switzerland, although this attitude differs from other countries. In the Netherlands, for example, forced medication is considered to be the most invasive type of coercive measure (31). That changes in attitudes towards different coercive measures and therefore a reduction of one kind of such measure can lead to an increase of another is a known phenomenon in empirical studies in general psychiatry (32).

### Limitations and Future Directions

As we pointed out in the *INTRODUCTION*, the present institution experienced major organizational changes in 2011 with minor adjustments in the following years. Efforts were made to adjust clinical processes with the goal to decrease coercive measures. The changes taken were established only in the institution itself but on all wards. So the interventions had impact on the prevalence of coercive measures on the “ward level” according to (17). The changes were not connected with an increase in the number of staff members which had shown to be relevant to reduce coercive measures (33). We did neither apply transformations in the architecture or interior design of the wards which has proven to be effective on the prevalence of coercion in general psychiatric care (34) nor did we establish a clearly defined special psychotherapeutic program which has proven effect even in forensic psychiatric care in the past (35).

A core intervention to reduce coercion was the obligation to repetitively visit trainings in de-escalating techniques. The positive effect on reducing coercive measures might be confounded by not only enhancing staff skills to manage imminent conflicts but also to increase sensitivity for situations with high risk of escalation, better communication between staff members and the knowledge about alternatives to seclusion or restraint. This might be understood as one aspect of a complex culture change within the present institution (36). Another aspect that might have contributed to decreasing rates of coercive measures might be the fact that the staff was committed to regulations and guidelines which include a debriefing after use of coercion. Although this was not practiced as a clearly defined “counselling intervention” there might be an effect by involving patients and staff into a reflecting process that has shown effectiveness (37).

What remains unclear is if the adjustments made are the direct cause for the reduction of coercive measures. With a retrospective study design it is not possible to detect direct causation. Neither can we isolate the factors that work or do not work, since we have introduced the organizational changes as a “package”. But it is known from the literature that “packages”—meant as complex interventions including different strategies on different organisational levels—can reduce rates of coercive measures even in forensic psychiatric facilities (38). It could be stated with caution that the changes initiated used four of six key components identified in the systematic review by Goulet et al. (39): leadership, training, post-seclusion and/or restraint review and prevention tools. But if those adjustments were directly linked to lowering seclusion, restraint and coerced medication cannot be verified.

Furthermore, strict empirical approaches with a control design would be desirable but not realistic to conduct due to ethical reasons. As coercive measures form an additional incision of personal rights, measures that are thought to reduce coercion may not be withheld from certain patients (*i.e.*, a control group).

It has to be taken into account that the rate of seclusion we reported might underestimate the real time a forensic patient experiences isolation from others because our study design detected only measures caused and initiated by individually stated risk of harm. Seclusion due to organizational reasons ordered for the whole group of patients on a ward was not ascertained (*e.g.*, major constructional works). A patient in restraint was also considered to be secluded as restraint is applied within seclusion (see above). The data on restraint can therefore be considered a more valid indicator for coercive measurement. The negative trend in restraint was also the most robust finding, while the negative trend in seclusion was only apparent on the descriptive but not inferential level. Due to the observational nature of the study, we were limited in the sample size, causing outliers to have a stronger impact on the overall data.

The study was performed in a forensic institution specialized in the treatment of offenders suffering from schizophrenia. The data covers a period of nine years which is a long timeframe compared to earlier studies. Though this study has its merit in reporting longitudinal data on coercive measures of an understudied sample, it must be noted that the data was obtained within a diagnostically homogenous group, limiting its generalizability. It is a common finding in general psychiatry that people suffering from schizophrenia are at higher risk of experiencing coercive measures as compared to other psychiatric patients (12). An implication of our study is that forensic inpatients with a history of criminal offences and who are suffering from schizophrenia can be treated without a high rate of coercive measures. It seems as if the milieu of a forensic institution with trained staff on dealing with potentially aggressive patients produces even fewer incidents of coercive measures as compared to treating patients suffering from schizophrenia in general psychiatric care. The factors underlying this effect of relatively low rates of coercive measures should be subject to future research.

One of the prominent issues inherent in the solution to violence in forensic settings is finding the balance between security and clinical treatment. An increase in security is often thought to undermine treatment. An important aspect of reducing coercive interventions is a possible increase of violence instead. A major limitation of the study is the lacking data about violent incidents. If the observed trend of a reduction of seclusion and restraint over a period of nine years was accompanied by a concomitant increase in violence against other patients and staff remains unclear. Future research should address this important clinical factor which is highly important to develop secure settings for patients and staff. It is also apparent from our study that only a minority of patients causes the majority of incidents of coercive measures. It should therefore also be a focus in future studies to identify and target this subgroup at risk for experiencing longer and repetitive coercive measures at an early stage.

## Data Availability Statement

The datasets for this manuscript are not publicly available because it contains possibly identifying information. Requests to access the datasets should be directed to NB at nathalie.brackmann@puk.zh.ch.

## Ethics Statement

Ethical approval was not required according to the cantonal ethics committee of Zurich, Switzerland (BASEC-Nr. Req-2019-00550).

## Author Contributions

SL, NB, AM, and EH contributed to the conception and design of the study. NB and AM organized the database. NB and AM performed the statistical analysis. SL wrote the first draft of the introduction and discussion. NB wrote the first draft of the *Method* and *Results* sections. All authors contributed to manuscript revision, read and approved the submitted version.

## Conflict of Interest

The authors declare that the research was conducted in the absence of any commercial or financial relationships that could be construed as a potential conflict of interest.

## References

[1] Hoff P Compulsory Interventions Are Challenging the identity of psychiatry. Front Psychiatry (2019) 10:783. 10.3389/fpsyt.2019.00783 31780962 PMC6851161

[2] Szmukler G Appelbaum PS Treatment pressures, leverage, coercion, and compulsion in mental health care. J Ment Health (2008) 17(3):233–44. 10.1080/09638230802052203

[3] Davison SE The management of violence in general psychiatry. Adv Psychiatr Treat (2005) 11(5):362–70. 10.1192/apt.11.5.362

[4] Hallett N Huber JW Dickens GL Violence prevention in inpatient psychiatric settings: Systematic review of studies about the perceptions of care staff and patients. Aggression Violent Behav (2014) 19(5):502–14. 10.1016/j.avb.2014.07.009

[5] Larue C Dumais A Boyer R Goulet M-H Bonin J-P Baba N The experience of seclusion and restraint in psychiatric settings: Perspectives of patients. Issues Ment Health Nurs (2013) 34(5):317–24. 10.3109/01612840.2012.753558 23663018

[6] Luciano M De Rosa C Sampogna G Del Vecchio V Giallonardo V Fabrazzo M How to improve clinical practice on forced medication in psychiatric practice: Suggestions from the EUNOMIA European multicentre study. Eur Psychiatry (2018) 54:35–40. 10.1016/j.eurpsy.2018.07.002 30118917

[7] Fiorillo A De Rosa C Del Vecchio V Jurjanz L Schnall K Onchev G How to improve clinical practice on involuntary hospital admissions of psychiatric patients: suggestions from the EUNOMIA study. Eur Psychiatry (2011) 26(4):201–7. 10.1016/j.eurpsy.2010.01.013 20965119

[8] Garriga M Pacchiarotti I Kasper S Zeller SL Allen MH Vazquez G Assessment and management of agitation in psychiatry: Expert consensus. World J Biol Psychiatry (2016) 17(2):86–128. 10.3109/15622975.2015.1132007 26912127

[9] Martin V Bernhardsgrütter R Goebel R Steinert T The use of mechanical restraint and seclusion in patients with schizophrenia: a comparison of the practice in Germany and Switzerland. Clin Pract Epidemiol Ment Health (2007) 3(1):1. 10.1186/1745-0179-3-1 17274830 PMC1797172

[10] Steinert T Lepping P Bernhardsgrütter R Conca A Hatling T Janssen W Incidence of seclusion and restraint in psychiatric hospitals: a literature review and survey of international trends. Soc Psychiatry Psychiatr Epidemiol (2010) 45(9):889–97. 10.1007/s00127-009-0132-3 19727530

[11] Noorthoorn E Lepping P Janssen W Hoogendoorn A Nijman H Widdershoven G One-year incidence and prevalence of seclusion: Dutch findings in an international perspective. Soc Psychiatry Psychiatr Epidemiol (2015) 50(12):1857–69. 10.1007/s00127-015-1094-2 26188503

[12] Hotzy F Theodoridou A Hoff P Schneeberger AR Seifritz E Olbrich S Machine Learning: An approach in identifying risk factors for coercion compared to binary logistic regression. Front Psychiatry (2018) 9:258. 10.3389/fpsyt.2018.00258 29946273 PMC6005877

[13] Keski-Valkama A Sailas E Eronen M Koivisto AM Lonnqvist J Kaltiala-Heino R The reasons for using restraint and seclusion in psychiatric inpatient care: A nationwide 15-year study. Nord J Psychiatry (2010) 64(2):136–44. 10.3109/08039480903274449 19883195

[14] Zhu X-M Xiang Y-T Zhou J-S Gou L Himelhoch S Ungvari GS Frequency of physical restraint and its associations with demographic and clinical characteristics in a Chinese psychiatric institution. Perspect Psychiatr Care (2014) 50(4):251–6. 10.1111/ppc.12049 24308920

[15] Hilton NZ Ham E Seto MC Assessment of risk for seclusion among forensic inpatients: Validation and modification of the risk of administrative segregation tool (RAST). Int J Offender Ther Comp Criminology (2019) 63(8):1424–45. 10.1177/0306624x18823621 31064294

[16] Günther MP Kirchebner J Lau S Identifying direct coercion in a high risk subgroup of offender patients with schizophrenia via machine learning algorithms. Front Psychiatry (2020) 11:415. 10.3389/fpsyt.2020.00415 32477188 PMC7237713

[17] Janssen WA van de Sande R Noorthoorn EO Nijman HL Bowers L Mulder CL Methodological issues in monitoring the use of coercive measures. Int J Law Psychiatry (2011) 34(6):429–38. 10.1016/j.ijlp.2011.10.008 22079087

[18] National Institute for Health and Care Excellence Violence and aggression: short-term management in mental health, health and community settings (NICE Guideline 10). National Collaborating Centre for Mental Health (2015). Available from: https://www.nice.org.uk/guidance/ng10. 40997203

[19] Hui A Middleton H Völlm B Coercive measures in forensic settings: findings from the literature. Int J Forensic Ment Health (2013) 12(1):53–67. 10.1080/14999013.2012.740649

[20] Crichton JH Defining high, medium, and low security in forensic mental healthcare: the development of the Matrix of Security in Scotland. J Forensic Psychiatry Psychol (2009) 20(3):333–53. 10.1080/14789940802542808

[21] Krammer S Arnold C Czuczor T Liebrenz M Bestimmung der Sicherheitsstufe forensisch-psychiatrischer Kliniken anhand der Matrix of Security [Determination of the security level of forensic psychiatric institutions using the Matrix of Security]. Schweiz Z für Krim. (2018) (1):4–11.

[22] Paavola P Tiihonen J Seasonal variation of seclusion incidents from violent and suicidal acts in forensic psychiatric patients. Int J Law Psychiatry (2010) 33(1):27–34. 10.1016/j.ijlp.2009.10.006 19962761

[23] Hui A Middleton H Völlm B The uses of coercive measures in forensic psychiatry: A literature review. In: Völlm B Nedopil N, editors. The Use of Coercive Measures in Forensic Psychiatric Care: Legal, Ethical and Practical Challenges. Springer International Publishing Switzerland (2016) p. 151–84.

[24] Jakovljević AK Wiesemann C Zwangsmaßnahmen in der forensischen Psychiatrie [Coercive procedures in forensic psychiatry]. Der Nervenarzt (2016) 87(7):780–6. 10.1007/s00115-015-4437-z 26482288

[25] Steinert T Martin V Baur M Bohnet U Goebel R Hermelink G Diagnosis-related frequency of compulsory measures in 10 German psychiatric hospitals and correlates with hospital characteristics. Soc Psychiatry Psychiatr Epidemiol (2007) 42(2):140–5. 10.1007/s00127-006-0137-0 17180296

[26] Flammer E Frank U Steinert T Freedom restrictive coercive measures in forensic psychiatry. Front Psychiatry (2020) 11:146. 10.3389/fpsyt.2020.00146 32194460 PMC7066111

[27] Almvik R Rasmussen K Woods P Challenging behaviour in the elderly—monitoring violent incidents. Int J Geriatric Psychiatry (2006) 21(4):368–74. 10.1002/gps.1474 16534771

[28] Lussier P Verdun-Jones S Deslauriers-Varin N Nicholls T Brink J Chronic violent patients in an inpatient psychiatric hospital:prevalence, description, and identification. Criminal Justice Behav (2010) 37(1):5–28. 10.1177/0093854809347738

[29] Vojt G Marshall LA Thomson LDG The assessment of imminent inpatient aggression: a validation study of the DASA-IV in Scotland. J Forensic Psychiatry Psychol (2010) 21(5):789–800. 10.1080/14789949.2010.489952

[30] Weizmann-Henelius G Suutala HJO Violence in a finnish forensic psychiatric hospital. Nord J Psychiatry (2000) 54(4):269–73. 10.1080/080394800448147

[31] Verlinde AA Noorthoorn EO Snelleman W van den Berg H Snelleman-van der Plas M Lepping P Seclusion and enforced medication in dealing with aggression: A prospective dynamic cohort study. Eur Psychiatry (2017) 39:86–92. 10.1016/j.eurpsy.2016.08.002 27992811

[32] Steinert T Noorthoorn EO Mulder CL The use of coercive interventions in mental health care in Germany and the Netherlands. A comparison of the developments in two neighboring countries. Front Public Health (2014) 2:141–. 10.3389/fpubh.2014.00141 PMC4173217 25309893

[33] Scanlan JN Interventions to reduce the use of seclusion and restraint in inpatient psychiatric settings: What we know so far a review of the literature. Int J Soc Psychiatry (2010) 56(4):412–23. 10.1177/0020764009106630 19617275

[34] Rohe T Dresler T Stuhlinger M Weber M Strittmatter T Fallgatter AJ Bauliche Modernisierungen in psychiatrischen Kliniken beeinflussen Zwangsmaßnahmen [Architectural modernization of psychiatric hospitals influences the use of coercive measures]. Der Nervenarzt (2017) 88(1):70–7. 10.1007/s00115-015-0054-0 26820456

[35] Goodness KR Renfro NS Changing a culture: a brief program analysis of a social learning program on a maximum-security forensic unit. Behav Sci Law (2002) 20(5):495–506. 10.1002/bsl.489 12239708

[36] Stead K Kumar S Schultz TJ Tiver S Pirone CJ Adams RJ Teams communicating through STEPPS. Med J Aust (2009) 190(S11):128–32. 10.5694/j.1326-5377.2009.tb02619.x 19485861

[37] Whitecross F Seeary A Lee S Measuring the impacts of seclusion on psychiatry inpatients and the effectiveness of a pilot single-session post-seclusion counselling intervention. Int J Ment Health Nurs (2013) 22(6):512–21. 10.1111/inm.12023

[38] Putkonen A Kuivalainen S Louheranta O Repo-Tiihonen E Ryynanen OP Kautiainen H Cluster-randomized controlled trial of reducing seclusion and restraint in secured care of men with schizophrenia. Psychiatr Serv (2013) 64(9):850–5. 10.1176/appi.ps.201200393 23771480

[39] Goulet M-H Larue C Dumais A Evaluation of seclusion and restraint reduction programs in mental health: a systematic review. Aggression violent Behav (2017) 34:139–46. 10.1016/j.avb.2017.01.019

